# Post-traumatic stress following social media war violence: role of empathy and emotional awareness in youth

**DOI:** 10.1192/bjo.2026.12014

**Published:** 2026-06-01

**Authors:** Daniel Schleicher, Irina Jarvers, Angelika Ecker, Stephanie Kandsperger, Romuald Brunner

**Affiliations:** Department of Child and Adolescent Psychiatry and Psychotherapy, University of Regensburg, Germany

**Keywords:** Post-traumatic stress, social media war violence, alexithymia, empathy, adolescence

## Abstract

This study investigated associations between exposure to war-related violent content on social media and indirect, media-related post-traumatic stress symptoms (PTSS) in a nationally representative sample of adolescents and young adults in Germany (*N* = 1860; *M*_age_ = 18.6 years). Participants completed an online survey assessing violent media exposure, PTSS, alexithymia, different facets of empathy and perceived burden related to current political crises. Regression analyses showed that higher alexithymia, greater somatic empathy, stronger perceived crisis-related burden, younger age and personal exposure to war-related events were significantly associated with higher PTSS, explaining 14.1% of the variance. In contrast, the frequency of violent social media exposure was not significantly related to symptom severity. The findings suggest that individual emotional processing characteristics and contextual burden are more strongly related to PTSS than mere exposure to violent social media content, highlighting the relevance of emotional awareness and bodily empathic reactivity in responses to online violence.

Ongoing political conflicts, such as the war in Ukraine and the Israel–Gaza war, are highly visible in the news and on social media. On social platforms, violent content showing torture, injury and death is increasingly accessible without safety mechanisms. Adolescents and young adults are considered particularly vulnerable given their developmental stage and high exposure to social media.^1^ Although direct trauma is a known risk factor for post-traumatic stress symptoms (PTSS), it remains unclear whether indirect exposure to violent media elicits similarly severe symptoms. Some studies report elevated stress levels and associations with PTSS in response to violent media exposure,^1–3^ but no study has systematically examined contributing factors such as emotional awareness (of one’s own emotions) or empathy (understanding others’ feelings). This study examines the association between social media exposure to war-related violence and indirect, media-related PTSS in a nationally representative sample of adolescents and young adults in Germany. Data were collected nationwide from 11 to 25 March 2025.

## Method

The survey was conducted via the Appinio online platform (Appinio GmbH, Hamburg, Germany; https://www.appinio.com/de/). Participants provided digital informed consent after being informed about the study’s objectives. Participation was voluntary and could be discontinued at any time. Participants residing in Germany at the time of assessment were recruited using predefined quotas (e.g. age, gender, region and socioeconomic status/education) to approximate a nationally representative sample. The authors assert that all procedures contributing to this work comply with the ethical standards of the relevant national and institutional committees on human experimentation, and with the Helsinki Declaration of 1975 as revised in 2013. All procedures involving human subjects/patients were approved by the Ethics Committee of the University of Regensburg (reference no. 24-3958-101, 20 December 2024). The study is registered in the German Clinical Trials Register (no. DRKS00036048, 21 February 2025). In addition to demographics, participants answered questions on exposure to violent images/videos of political crises and armed conflicts on social media, including depictions of injury, torture, killing or hostage-taking (e.g. frequency, platforms, personal impact). PTSS (intrusions, hyperarousal, avoidance and sleep problems) were assessed in relation to violent media exposure. Items were explicitly anchored to this context (e.g. ‘How do these images or videos affect you?’ – ‘The images keep coming back to my mind unintentionally’). In addition, general crisis-related burden was assessed (e.g. ‘Do you feel burdened by ongoing political crises and conflicts in your daily life, such as thinking about them frequently or worrying a lot?’). Emotional awareness was assessed with the Perth Alexithymia Questionnaire–Short Form (PAQ-S).^4,5^ Furthermore, affective (feeling others’ emotions: ‘I often feel what other people are feeling’), cognitive (understanding others’ emotions: ‘I often understand what other people are feeling’) and somatic empathy (physically experiencing others’ emotions: ‘I often feel in my body, for example a faster heartbeat or sweating, what other people are feeling’) were measured (adapted from Raine and Chen^6^ and Schleicher et al^7^) (Table 1).

**Table 1:** Descriptive statistics (upper section) and regression analysis predicting PTSS (lower section)

Variable	*n* (%)	Variable	*n* (%)
Gender		Violent media frequency	
Female	924 (49.70)	Daily	383 (20.60)
Male	936 (50.30)	Weekly	553 (29.70)
Educational/occupational status		Several times per month	411 (22.10)
Special needs school	17 (0.90)	Once per month	210 (11.30)
Lower secondary school	140 (7.50)	Every 2–4 months	111 (6.00)
Intermediate secondary school	243 (13.10)	Every 5–6 months	51 (2.70)
Academic secondary school	483 (26.00)	Less often	141 (7.60)
Vocational/technical school	146 (7.80)	Violent media exposure	
Vocational training/apprenticeship	248 (13.30)	Intentional (actively searched)	189 (10.20)
University studies	218 (11.70)	Unintentional (in feed, sent to me)	1051 (56.50)
Employed	192 (10.30)	Both	470 (25.30)
Self-employed	28 (1.50)	Unsure	150 (8.10)
Not currently employed	116 (6.20)	Violent media platform	
Other	29 (1.60)	Instagram	1031 (55.40)
Nationality/citizenship		TikTok	1194 (64.20)
Germany	1749 (83.40)	Snapchat	292 (15.70)
Turkey	49 (2.30)	YouTube	618 (33.20)
Russia	41 (2.00)	WhatsApp	368 (19.80)
Poland	27 (1.30)	Darknet	95 (5.10)
Other	230 (11.00)	X/Twitter	23 (1.20)
Religion		Reddit	11 (0.60)
Christianity	1016 (54.60)	Telegram	5 (0.30)
Islam	273 (14.70)	Television / News	10 (0.50)
Atheism	249 (13.40)	Other	12 (0.60)
No religious affiliation	82 (4.40)	Not sure	82 (4.40)
Judaism	27 (1.50)	PTSS	
Hinduism	16 (0.90)	Intrusions	404 (21.70)
Buddhism	14 (0.80)	Hyperarousal	274 (14.70)
Prefer not to say	123 (6.60)	Avoidance	486 (26.10)
Other	60 (3.20)	Sleep problems/nightmares	197 (10.60)
War exposure			Mean (s.d.)
Not at all	1392 (74.80)	Number of PTSS	0.73 (0.79)
Close relations in war zone	389 (20.90)	Burdened by crises	2.55 (0.84)
Has personally been in war zone	118 (6.30)	Alexithymia (PAQ-S)	24.63 (6.91)
		Affective empathy	2.90 (0.84)
		Cognitive empathy	2.98 (0.84)
		Somatic empathy	2.43 (0.92)

Dependent variable	Predictors	*B*	s.e.	95% CI *B*		*β*	*t*	*p*	*R* ^2^	Adj. *R* ^2^
				Lower bound	Upper bound					
PTSS	Age	**−0.03**	**0.01**	**−0.05**	**0**	**−0.05**	**−2.27**	**0.023**	0.14	0.14
	Gender	0	0.04	−0.07	0.07	0	0	0.998		
	Alexithymia (PAQ-S)	**0.01**	**0**	**0**	**0.01**	**0.08**	**3.50**	**<0.001**		
	Violent media frequency	−0.01	0.01	−0.03	0.01	−0.02	−0.96	0.335		
	War exposure (close relations)	**0.14**	**0.05**	**0.06**	**0.23**	**0.07**	**3.19**	**0.001**		
	War exposure (self)	**0.20**	**0.07**	**0.06**	**0.34**	**0.06**	**2.73**	**0.006**		
	Burdened by crises	**0.24**	**0.02**	**0.20**	**0.29**	**0.26**	**11.13**	**<0.001**		
	Affective empathy	0.02	0.02	−0.03	0.06	0.02	0.76	0.449		
	Cognitive empathy	0	0.02	−0.05	0.04	0	−0.11	0.913		
	Somatic empathy	**0.09**	**0.02**	**0.05**	**0.13**	**0.11**	**4.61**	**<0.001**		

PTSS, post-traumatic stress symptoms; PAQ-S, Perth Alexithymia Questionnaire–Short Form; *B*, unstandardised coefficient; *β*, standardised coefficient.

Multiple responses per person are possible for ‘citizenship’ and ‘violent media platform’. Response range for ‘burdened by crises’ and empathy scales: 1–4.

Significant predictors are marked in bold font.

Of 5994 invited participants, 1961 were pre-excluded (e.g. no consent) and 720 dropped out. Following data collection, 1313 cases were removed (977 for data quality, 336 randomly removed by the survey provider after quota fulfilment), yielding 2000 participants. The final sample comprised 1860 participants (*M*_age_ 18.60, s.d._age_ 1.58, range 16–21) following exclusion of 135 with no violent media exposure and 5 gender-diverse participants due to small group size.

A multiple linear regression examined predictors of PTSS. The dependent variable was the number of endorsed PTSS; independent variables included gender, age, war exposure, violent media frequency, alexithymia, empathy and perceived burden from current crises. In addition, hierarchical (nested) regression models were computed to examine the incremental contribution of predictor blocks (see Supplementary Material 1 available at https://doi.org/10.1192/bjo.2026.12014).

## Results

The regression model was significant, explaining 14.1% of the variance in PTSS (*F*(10, 1859) = 30.32, *p* < 0.001). Significant predictors were higher alexithymia, younger age, war exposure (of oneself or close relations), greater somatic empathy and higher perceived burden from ongoing crises (Table 1). In contrast, violent media frequency did not emerge as a significant predictor. Hierarchical regression analyses showed that conflict-related exposure variables and affective traits accounted for incremental variance in PTSS beyond demographics (see Supplementary Material 1 for nested models and a visual representation of the regression coefficients).

## Discussion

In this large sample of adolescents and young adults, PTSS were more strongly linked to individual and contextual factors than to violent media exposure itself. Lower emotional awareness, greater somatic empathy and stronger perceived crisis-related burden were linked to greater symptom endorsement, suggesting that heightened bodily empathic reactivity, in combination with difficulties in identifying and articulating one’s own emotional states, may be associated with distress responses to crisis-related content. Additionally, younger participants and those personally affected by crises, either directly or through family and acquaintances, reported more symptoms, suggesting an important role of developmental stage and proximity to traumatic events. With regard to age, the present findings overlap with those reported by Slone et al,^1^ who found positive associations between exposure to armed conflict-related content on the internet and post-traumatic symptoms only among adolescents up to 18 years of age, but not among young adults aged 20–26 years. This pattern may be partly attributable to developmental differences, because older individuals may be more likely to engage in cautious and reflective appraisal of social media content and to draw on more stable emotion regulation and mentalisation capacities.^1,8^

Limitations of our study include reliance on self-reported data, the absence of a detailed content analysis of the viewed videos and the assessment of psychological symptoms rather than distinct psychiatric diagnoses. In addition, the cross-sectional design does not allow us to distinguish between state- and trait-like aspects of emotional processing, or to disentangle the influence of prior developmental experiences. Because the PAQ-S, for example, does not assess the developmental history of these characteristics, findings should be interpreted as reflecting current levels of emotional awareness. Moreover, social or peer-related contextual influences were not assessed and may also contribute to similarities in emotional processing. Strengths include a large, nationally representative German sample focusing on adolescents and young adults, as well as provision of the first systematic examination of empathy- and alexithymia-related factors in the emotional processing of violent social media content. Future research should incorporate more detailed assessments of trauma exposure, examine potential interaction effects and use approaches that better disentangle the relative contributions of different predictor domains, to further clarify the roles of personal experiences and media-related factors.

Looking ahead, structural efforts to strengthen protective measures and media literacy will be essential, and clinical research and interventions should also address personality and social factors to support youth mental health.^9^ In this context, the association between emotional processing of violent social media content and distress underscores the relevance of empathy and alexithymia for clinical assessment, psychoeducation and interventions targeting emotional awareness, coping and emotion regulation.

## Supporting information

10.1192/bjo.2026.12014.sm001 Schleicher et al. supplementary material Schleicher et al. supplementary material

## Data Availability

The data are available from author D.S. upon reasonable request.

## Supplementary material

The supplementary material is available online at https://doi.org/10.1192/bjo.2026.12014
