# Supplementary material for: Post-traumatic stress following social media war violence: role of empathy and emotional awareness in youth
Source: BJPsych Open. 2026 Jun 1;12(4):e150. doi: 10.1192/bjo.2026.12014 (PMC13237116; doi:10.1192/bjo.2026.12014)
Supplement: Schleicher et al. supplementary material [file S2056472426120146sup001.pdf]

Supplementary Material 1. Hierarchical regression models predicting PTSS

Hierarchical (nested) linear regression models were conducted to examine the incremental contribution of predictor blocks to PTSS. Demographic variables (age, sex) were entered in Model 1, conflict-related variables (war exposure, perceived burden, violent media frequency) in Model 2, and affective traits (alexithymia, cognitive, affective, and somatic empathy) in Model 3.

	**Model 1 (Demographics)**		**Model 2 (+ Conflict variables)**		**Model 3 (+ Affective traits)**	
**Predictors**	***B* (SE)**	**β**	***B* (SE)**	**β**	***B* (SE)**	**β**
Age	-0.02 (0.01)	-0.03	**-0.02 (0.01)***	**-0.05**	**-0.03 (0.01)***	**-0.05**
Sex	0.00 (0.04)	0.00	0.01 (0.04)	0.00	0.00 (0.04)	0.00
Burdened by crises	-	-	**0.27 (0.02)*****	**0.29**	**0.24 (0.02)*****	**0.26**
Violent media frequency	-	-	-0.02 (0.01)	-0.04	-0.01 (0.01)	-0.02
War exposure (myself)	-	-	**0.24 (0.07)*****	**0.07**	**0.20 (0.07)****	**0.06**
War exposure (close relations)	-	-	**0.17 (0.05)*****	**0.09**	**0.14 (0.05)****	**0.07**
Alexithymia (PAQ-S)	-	-	-	-	**0.01 (0.00)*****	**0.08**
Affective empathy	-	-	-	-	0.02 (0.02)	0.02
Cognitive empathy	-	-	-	-	0.00 (0.02)	0.00
Somatic empathy	-	-	-	-	**0.09 (0.02)*****	**0.11**

Model 1: R^2 = .00, Adj. R^2 = .00, F(2, 1859) = 1.09, *p* = .335
Model 2: R^2 = .12, ΔR^2 = .12, Adj. R^2 = .12, F(6, 1859) = 41.55, *p* < .001
Model 3: R^2 = .14, ΔR^2 = .02, Adj. R^2 = .14, F(10, 1859) = 30.32, *p* < .001

Forest plot of regression coefficients (*B*) from Model 3 (95% confidence intervals)

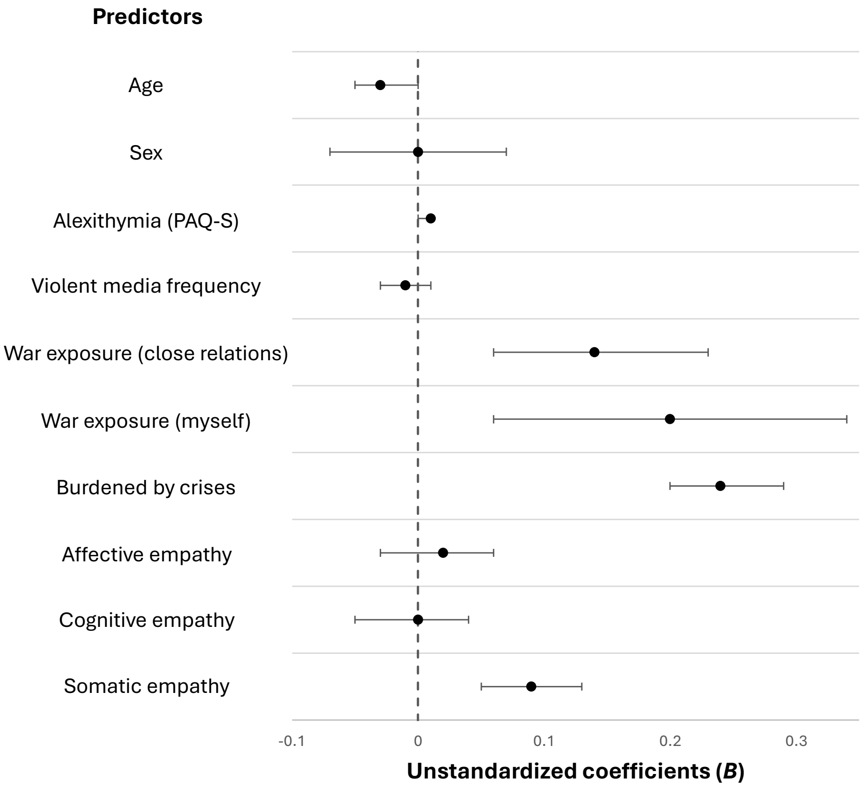

Note. PTSS = Posttraumatic stress symptoms. *B* = unstandardized coefficient. SE = standard error. β = standardized coefficient. ΔR^2 reflects the change in explained variance compared to the previous model. *p < .05, **p < .01, ***p < .001. Significant predictors are marked in bold font. Response range for "burdened by crises" and empathy scales: 1–4. PAQ-S = Perth Alexithymia Questionnaire–Short Form.
